# 

**Published:** 1855-11

**Authors:** 


### CONTENTS.

### ORIGINAL COMMUNICATIONS.

PAGE.
ARTICLE I.—Report of Cases in the Medical Clinic. By J. G. WESTMORELAND, M. D., Professor of Materia Medica and Medical Jurisprudence in the Atlanta Medical College.......... 129
ARTICLE II.—A Case of Hysteria complicated with Mania-a-Potu. By THOS. S. DENNY, M. D., Atlanta, Georgia.......... 132
ARTICLE III,—Physiological and Pathological Observations connected with the effects of Alcoholic Drinks upon the Liver. By E. HILLYER, M. D., Atlanta, Georgia.......... 133
ARTICLE IV.—Paracentesis Thoracis. Translated from the French by W. F. WESTMORELAND, M. D., Professor of Principles and Practice of Surgery in the Atlanta Medical College.......... 137

### SELECTIONS.

The Causes of Hemorrhage from the Unimpregnated Uterus.......... 146
On Sterility depending on certain diseased states of the Lining Membrane of the Womb—its treatment and cure.......... 152
Considerations on the Reciprocal Influence of the Physical Organization and Mental Manifestations.......... 157
Account of a Case of Catalepsy and Spontaneous Somnambulism.......... 166
On Uterine Pains and Hemorrhage after Delivery.......... 173
On the Treatment of Cholera with Strychnia.......... 180
Editorial and Miscellaneous.......... 183

